# Facing fears in virtual worlds: A systematic review and meta-analysis on immersive VR therapy for children and adolescents with social anxiety and related disorders

**DOI:** 10.1007/s00787-025-02945-w

**Published:** 2026-01-21

**Authors:** J. F. Baschab, E. Moehler, J. Hussong

**Affiliations:** 1 Department of Child and Adolescent Psychiatry, Saarland University, 66421 Homburg, Germany; 2 Child and Adolescent Psychiatry, Saarland University Medical Center, 66421 Homburg, Germany

**Keywords:** Systematic review, Meta-analysis, Virtual reality exposure therapy, Children, Adolescents, Anxiety disorders, Social anxiety

## Abstract

Anxiety disorders are common mental health issues in children and adolescents. Virtual reality (VR) offers promising opportunities for exposure-based interventions in immersive and controlled environments. This systematic review aimed to evaluate the effectiveness of VR-based exposure therapies for anxiety disorders and related symptoms in children and adolescents aged 4 to 18 years. A secondary objective was to assess user experience and adherence, where reported. A systematic search of six databases (Web of Science, ScienceDirect, PubPsych, EBSCOhost, PubMed, Cochrane Library) was conducted from January 1, 2015, to April 11, 2025 (last date of search), in title and abstract fields. Studies were included if they involved immersive VR-based exposure interventions targeting anxiety disorders (including social anxiety, generalized anxiety disorder, separation anxiety, or related symptoms) in participants aged 4 to 18 years. A narrative synthesis was conducted, and a random-effects meta-analysis was performed on subjective units of distress (SUDs) from eligible studies. Five out of 466 screened studies met the inclusion criteria. VR-based exposure therapy was associated with significant reductions in anxiety-related symptoms, particularly in social and public speaking anxiety. The meta-analysis of SUD ratings showed a large pre-post effect (Hedges’ *g* = − 1.51, (95%CI [− 2.20, − 0.82], *p* < .001), though heterogeneity was high (*I*² = 81.0%), and number of included studies small (*k* = 3). VR-based interventions appear to be effective in reducing anxiety symptoms in youth. However, the small number of studies, limited sample sizes, and methodological heterogeneity highlight the need for further research using standardized outcome measures.

## Introduction

### Anxiety disorders in children and adolescents

Anxiety disorders are among the most prevalent mental health conditions in children and adolescents. These include generalized anxiety disorder (GAD), separation anxiety disorder, social anxiety disorder (SAD), specific phobias, and panic disorder. In 2019, approximately 14% of individuals aged 10 to 19 worldwide were affected by mental health conditions. 4.4% of children aged 10 to 14 and 5.5% of adolescents aged 15 to 19 were diagnosed with anxiety disorder [1, 2]. These rates increased substantially during the COVID-19 pandemic, with prevalence rates rising to 13.0% among those under 18 years old [3]. If left untreated, anxiety disorders can lead to significant impairments in academic performance, social interactions, and family life. Furthermore, they are associated with an increased risk of developing chronic mental health issues in adulthood, including depression, substance use disorders, and other psychiatric illnesses [4, 6]. Although a wide range of digital and non-digital treatments is available, many young people remain undiagnosed or do not receive adequate care [2, 7, 8]. Barriers to access include not only a global shortage of mental health professionals [2, 9], but also individual factors such as the fear of stigmatization [10], negative attitudes toward treatment [11], and prior negative treatment experiences [12]. Although effective treatments exist, these barriers often limit their uptake and reach.

### Current treatment approaches and limitations

Cognitive behavioral therapy (CBT) is widely considered the gold standard for treating anxiety disorders in children and adults [13, 16]. A core component of CBT for anxiety is exposure therapy, which involves step for step confrontation with the feared situation or stimuli in a safe and controlled environment. Exposure aims to reduce anxiety by promoting habituation and disconfirming catastrophic beliefs, as well as by reducing avoidance [17, 18]. However, implementing exposure therapy in real life can be challenging. Feared situations are difficult to recreate, real-world situations are hard to control, and often initial symptoms worsen, which leads to high dropout rates and low engagement among patients [19, 21]. These limitations have motivated research into alternative formats that preserve therapeutic principles of exposure while reducing its practical barriers.

## Virtual reality as a therapeutic tool

A promising alternative format to traditional in vivo exposure is virtual reality exposure therapy (VRET). VRET uses immersive virtual reality (VR) environments specifically designed for exposure-based interventions. In VRET, patients are systematically exposed to anxiety-provoking situations within a virtual setting that allows repeated, safe, and graded exposure [22, 23]. Compared to imaginal or even in vivo exposure, VR can simulate real-world environments with high sensory realism and interactivity while remaining controllable for the therapist [24, 26]. It also enables access to scenarios that are logistically difficult or ethically challenging to reproduce in vivo (e.g., public speaking, social interactions, school settings).

Initial findings show that the efficacy of VR exposure is comparable to traditional in vivo exposure [27, 40]

–32]. Beyond this, VR-based interventions may enhance ecological validity, reduce logistical constraints, and increase patient engagement. Children and adolescents, in particular, may find VR appealing due to its interactive and game-like nature [33, 37].

Yet, most studies to date have focused on adults [25, 29, 30, 36, 38]. In adult populations, VR-based exposure therapy has been shown to be effective for social anxiety and other disorders. Meta-analytic evidence indicates large effect sizes when comparing VRET to waitlist controls (Opriş et al. [41]: *d* = 1.01, 95% CI [0.69, 1.33]; Kampmann et al. [42], 2016: *g* = 0.82, 95% CI [0.13, 1.51], and Chesam [43], 2016: *g* = 0.82, 95% CI [0.49, 1.15]). Comparisons to standard in vivo or imaginal exposure across studies revealed negligible and non-significant differences (Opriş et al. [41]: *d* = 0.13, 95% CI [− 0.11, 0.38], Kampmann et al. [42], *g* = − 0.24, 95% CI [− 0.71, 0.23], and Chesam [43], 2016: *g* = − 0.01, 95% CI [− 0.30, 0.28], indicating comparable efficacy. These meta-analyses included both small and larger studies and highlight the risk of publication bias favoring significant results. While most studies focused on adults with social anxiety disorder, generalizability to younger populations remains unclear and the questions arise, whether findings from adult populations can be generalized to younger age groups [29, 39, 44, 45].

Moreover, factors such as presence, i.e. the perceived feeling of being inside the simulation, and cybersickness may influence treatment effectiveness, as well as user adherence and user experience [45, 47]. Adherence refers to objectively measurable behaviors, such as session completion or therapist protocol compliance [48], while user experience captures subjective aspects, including usability, engagement, realism, and satisfaction [49]. Both constructs are critical for evaluating feasibility and long-term applicability of VR interventions in clinical practice.

## This study

To address these gaps, the present systematic review synthesized evidence on the effectiveness and user experience of virtual reality (VR) interventions for treating anxiety disorders in children and adolescents aged 4 to 18 years. The review focused on generalized anxiety disorder (GAD), separation anxiety disorder, social anxiety disorder (SAD), and social phobia. While the review targeted anxiety disorders broadly, the evidence base primarily consisted of studies on social anxiety and public speaking anxiety.

The timeframe was restricted for studies published between January 1, 2015, and April 11, 2025, to capture the period in which modern immersive VR systems became widely available and clinically applicable. Earlier interventions often relied on desktop-based systems or less immersive technology, which limits comparability to current applications using head-mounted displays with high resolution and interactivity. By focusing on the most recent decade, this review emphasizes evidence derived from contemporary VR technology that is more representative of current clinical practice. Nonetheless, restricting the timeframe may exclude earlier formative studies, which is acknowledged as a limitation.

The objectives were:

1. To evaluate the effectiveness of VR-based exposure therapies for anxiety disorders and related symptoms in children and adolescents aged 4–18 years, drawing on studies published from 2015 to 2025.
2. To assess user experience, including engagement and adherence, where reported.

These objectives also encompass providing an overview of current literature, identifying research gaps, and offering recommendations for future research in VR-based interventions in youth.

## Methods

This review was conducted using the PRISMA (Preferred Reporting Items for Systematic Reviews and Meta-Analyses) guidelines [50, 51]. The review protocol was registered in the PROSPERO international database on May 20, 2025 (CRD420251028708). The aim was to evaluate the effectiveness of immersive virtual reality (VR) interventions for anxiety disorders in children and adolescents, and to assess user experience, including engagement and adherence. Studies published between January 1, 2015, and April 11, 2025, were included to capture recent technological developments in VR interventions.

## Eligibility criteria

To be included in this systematic review, studies had to meet the following criteria:

- The study population consisted of children or adolescents aged 4 to 18 years who had either received a clinical diagnosis of an anxiety disorder (generalized anxiety disorder, separation anxiety disorder, social anxiety disorder, social phobia or exhibited clinically significant anxiety-related symptoms (e.g., fear of public speaking).
- Clinically significant anxiety-related symptoms were defined as either a formal diagnosis of an anxiety disorder or elevated scores on validated anxiety questionnaires indicating functional impairment.
- The intervention involved the use of virtual reality (VR) and included an immersive VR system providing a realistic and interactive experience.
- The study was published on or after January 1, 2015, to ensure an up-to-date overview of the last 10 years of research.
- Studies using any research design were eligible, including quantitative, qualitative, and mixed methods approaches, as well as various comparator designs (e.g., comparisons of VR with non-VR interventions or pre-post analyses).
- VR interventions could be implemented as stand-alone treatments or in combination with other therapeutic approaches.
- The study had to report on the effectiveness of the intervention and be published in a peer-reviewed journal.
- Only studies written in English or German were included.

### Search strategy and Article selection

A systematic search was conducted across six scientific research databases between January 1, 2015, and April 11, 2025: Web of Science, ScienceDirect, PubPsych, EBSCOhost (via the Saarland University interface), Cochrane Library and PubMed. The following keywords were used to search the databases: (“virtual reality” or “immersive virtual reality” or “VR-based therapy”) and (“anxiety disorder” or “social anxiety” or “social phobia” or “generalized anxiety disorder” or “GAD” or “separation anxiety”) and (“child” or “adolescent” or “youth” or “young people”) and (“exposure therapy” or “cognitive behavioral therapy” or “CBT” or “training” or “game”).

Searches were conducted in the title and abstract fields. Filters applied included publication date (2015–2025), language (English, German), and participant age (4–18 years). Grey literature (e.g., dissertations, preprints, unpublished data) was not included in the search, which may contribute to publication bias.

#### Study selection

Search results from all databases were de-duplicated, and the remaining articles and abstracts were screened based on their title and abstracts by one reviewer (JB). Subsequently, two reviewers independently assessed the full texts of potentially relevant studies (JB and JH). Disagreements were resolved through discussion and consensus, based on the predefined eligibility criteria. JB is a clinical psychologist with prior training in conducting systematic reviews, JH is a child and adolescent psychotherapist and clinical psychologist, holds a PhD, and has extensive expertise in systematic review methodology.

#### Data extraction

Data were extracted using standardized table including the following information: author(s), year, country, study design, peer-review status, participant age and sample size, diagnoses, recruitment setting, type and description of VR intervention, combination with CBT, session duration/frequency, presence of gamification elements, type of control group, measurement tools, timepoints assessed, main results, and relevant notes.

#### Quality assessment

As this review included both randomized controlled trial (RCT) and nonrandomized controlled studies, the Mixed Methods Appraisal Tool (MMAT) was used to assess the methodological quality of the included studies [52]. Each study was evaluated according to the relevant MMAT criteria based on its design.

#### Data synthesis

A narrative synthesis approach [53] was applied to summarize findings across studies. This involved grouping studies by outcomes, highlighting patterns, and interpreting results in relation to the review questions. To evaluate the effect of VR interventions on subjective distress, a random effects meta-analysis was conducted with R (Version 4.5.0) using the meta package [54]. Pre and post data from eligible studies were used, with effect sizes expressed as Hedges’ *g*. Random-effects models (REML) were applied, and heterogeneity was assessed using *I*² statistics. Sensitivity analyses were not conducted because only three studies were included, which limits the feasibility and interpretability of such analyses, in line with the PRISMA 2020 guidelines [50, 51].

## Results

### Study selection and quality assessment

**Fig. 1:**
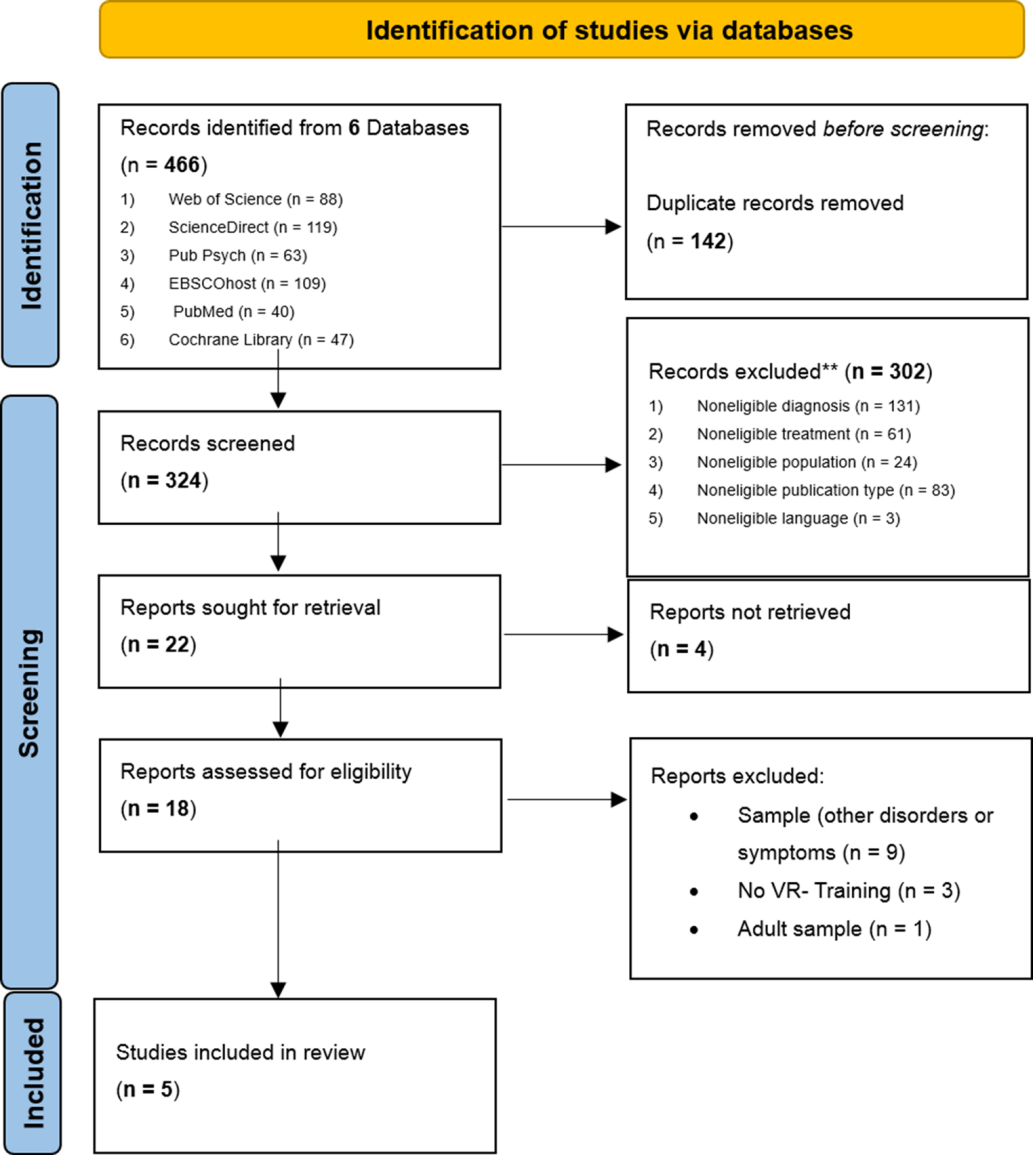
*Flowchart of the systematic research results. Note.*The diagram illustrates the identification, screening, and selection process of studies investigating immersive virtual reality-based interventions for anxiety-related symptoms in children and adolescents. Of 466 records initially identified, 5 studies were included in the final review after applying eligibility criteria. VR= virtual reality

The systematic database search yielded 466 articles (Fig. 1). After removing duplicate entries, 324 (69.5%) remained. Following title and abstract screening and full-text review, five studies (1.1%) met the eligibility criteria and were included in the systematic review. Several full-text articles were excluded despite initially appearing to meet inclusion criteria. Reasons for exclusions were that participants did not have a clinically confirmed anxiety disorder or related symptoms (*n* = 9), non-immersive VR interventions (*n* = 3) or participants outside the age range of 4–18 years (*n* = 1).

A detailed overview of the quality assessment results is presented in Appendix Table 6. Randomization was reported in three out of five studies [55, 57]. All randomized studies reported comparable baseline group analyses and complete outcome data. However, blinding of the outcome assessors was not mentioned in any of the included studies. In the quantitative non-randomized studies [58, 59], participants were representative of the target population, used appropriate outcome measures, clearly described the intervention procedures, and reported complete outcome data. Potential cofounders were only partially addressed or not reported in the study of Beele et al. [58], whereas Kahlon et al. [59] appropriately accounted for them in their analysis. Both studies confirmed that the intervention was delivered as intended.

### Study design and participants

An overview of study designs and comparisons of conditions is provided in Table 1. Three out of five studies [55, 57] were randomized controlled studies. The comparison groups in these studies included verbal exposure, imaginal exposure, VR based exposure, and combinations with only exposure program, online psychoeducation, or a waitlist control with additional online exposure. All studies implemented pre- and posttest assessments of participant outcomes. Additionally, two studies [56, 59] included follow-up assessments, with the longest follow-up occurring three months after the post-intervention assessment.

Across all studies, a total of 210 participants were recruited. The sample size ranged from 10 to 100 participants, with a mean sample size of 42 per study. Participants were from Norway (*n* = 132), the USA (*n* = 65) and Germany (*n* = 13). Participants were aged 7 to 18 years. Samples were predominantly female, with an average proportion of 75.3% (SD = 10.3%; range [60.0% − 84.0%]).

Most participants were diagnosed with social anxiety disorder (SAD) and/or generalized anxiety disorder (GAD) or reported severe public speaking anxiety (PSA) symptoms. All diagnoses and symptoms were determined using validated diagnostic instruments.

Beele et al. [58] included participants based on school-related trait anxiety (AFS) [AFS; 60], social anxiety symptoms [DISYPS-III; 61], and state anxiety (SUDs) during VR exposure. Biggs et al. [55] included children with a clinical anxiety disorder diagnosed via MINI-KID [62] and Spence Children’s Anxiety Scales [SCAS; 63], Kahlon et al. [56, 59] included adolescents with elevated public speaking anxiety on the PSAS [64]. Whiteside et al. [57] included children diagnosed with anxiety disorders according to DSM-IV-TR criteria using structured clinical interviews.

**Table 1:** Description of participants and research designs

Study	Country	Total sample size	Included sample size	Included sample mean age (years; SD)	Included sample age range (years)	Included sample percentage of female participants	Study design	Treatment conditions (sample size)	Measurements
Beele et al. [58]	Germany	13	10	15.7 (0.9)	14–17	60.0%	Quantitative non-randomized study	VRET (10)	Pre- and posttest measurement
Biggs et al. [55]	United States	45	45	13.2 (2.8)	7–17	84.4%	Quantitative randomized controlled study	VRET S 1 (45), verbal exposure S 1 (45), VRET S 2 (41), verbal exposure S 2 (41)	Pre- and posttest measurement
Kahlon et al. [59]	Norway	32	27	14.2 (0.6)	13–16	78.0%	Quantitative non-randomized study	VRET (27)	Pre-post measurement and 1- and 3-month FU
Kahlon et al. [56]	Norway	100	73	14.2* (1.0)*	13–16*	84.0%*	Quantitative randomized controlled study	VRET + no additional intervention (16); VRET + online exposure (16); online psychoeducation + online exposure (26); WL + online exposure (15)	Pre-post measurement and 3-month FU
White-side et al. [57]	United States	20	20	13.8 (2.9)	8–18	70.0%	Quantitative randomized controlled study	VRET (45), verbal exposure (45)	Pre-Post

Included sample: in analysis included sample. VRET: Virtual reality exposure therapy. S: session. FU: Follow-Up. * = values only for total sample reported

### Details of the VR interventions

All included studies implemented unique VR-based exposure therapies (see Table 2). The VR hardware included head-mounted devices (HMDs), smartphones, or VR glasses. The software immersed participants in interactive VR environments simulating social situations. The number of VR sessions ranged from 1 to 15, with session durations varying between 30 and 90 min. All interventions were delivered under the supervision of healthcare professionals, ensuring therapeutic support and adherence to protocol. The VR exposure components were either integrated into cognitive behavioral therapy (CBT) frameworks or offered as stand-alone treatments.

**Table 2:** Virtual reality interventions

Study	Virtual environments and exposures	Hardware	Treatment (duration)
Beele et al. [58]	School environment: street in front of the school, freely exploring the school building, sitting in the classroom, exercises before the classroom	Head-mounted display (VR-glasses); headphones (VIVE Pro)	VR exposure sessions, 5 sessions, 60 min each, four weeks
Biggs et al. [55]	Standing in a hallway at school, answering questions in class, giving presentations in class, taking a test, conversations with peers, asking teacher to get to bathroom, …	Pico Goblin Headset	VR exposure and imaginal exposure, 2 sessions, 60 min each, one week
Kahlon et al. [59]	Exposure tasks in a classroom with other students sitting at their desk; empty classroom and lobby as entry	Apple iPhone 7; high-end Cardboard-type VR headset	VR exposure, one session, 90 min
Kahlon et al. [56]	Conducting speeches in front of a virtual audience in a classroom	Oculus Quest 1	At least three weekly VRET sessions (15 possible levels), 30 to 60 min each, three weeks
Whiteside et al. [57]	Sitting in a classroom with other students when learning that the participant did poorly in on an assignment; Teacher announcing that they will be collecting an assignment, participant knows that the grade will be bad	Google Pixel Android Smartphone; head mounted headset (Daydream Viewer); handheld motion controller	VR exposure and verbal exposure, one session, 60 min

VR: Virtual reality

In Beele et al. [58], participants completed five therapist-led VR sessions over four weeks, which included phases such as acclimatization, exploration of the school building, classroom exercises, and stress tasks that were repeated until self-reported anxiety decreased by approximately 4%. Biggs et al. [55] combined VR and imaginal exposure across two 60-minute sessions in one week, with additional daily homework assignments for parent-supported practice at home, including classroom presentations, peer interactions, and typical school activities. Kahlon et al. [59] (2019) implemented a single 90-minute therapist guided VR session in a classroom scenario, with post-treatment follow-ups conducted one and three months later to assess long-term effects. Kahlon et al. [56] (2023) developed a self-guided home-based VR intervention over three weeks, with at least three weekly sessions of 30 to 60 min each, structured around fifteen gamified levels, speech tasks, audience interaction, and performance feedback, complemented by online psychoeducation. Whiteside et al. [57] conducted a single 60-minute therapist-guided session combining VR and verbal exposure, in which participants engaged in classroom-based scenarios involving assignments and peer of teacher interactions, continuously rated their anxiety, and completed post-session interviews.

These differences in session frequency, delivery format (home-based vs. therapist-led), gamification, and follow-up procedures illustrate the variability in VR implementation and may help explain differential outcomes across studies.

### Effectiveness of VR exposure on social and school-related anxiety

Across the five included studies (see Table 3), various outcome measures were used to evaluate the effectiveness of VR exposure on social and school-related anxiety symptoms. Beele et al. [58] reported a significant reduction in social anxiety symptoms [DISYPS; 61] and in subjective units of distress [SUD; 65]. Improvements in school-related anxiety were moderate, with a significant reduction in school reluctance [AFS; 60], but no significant changes in test anxiety and general anxiety. Biggs et al. [55] observed significant reductions in SUDs. Kahlon et al. [56, 59] found large reductions in public speaking anxiety symptoms [PSAS; 64]. Additionally, Kahlon et al. [56] reported a small but significant improvement in social phobia symptoms [SPS-6; 66], but no significant changes in social interaction anxiety symptoms [SIAS-6; 66]. Whiteside et al. [57] also found a large effect in reducing SUDs.

**Table 3:** Measurements of social anxiety symptoms (before/after VR intervention)

Study and measures	*N*	VR intervention outcomes	Effect sizes/test statistics	*p*-values
Beele et al. [58]	10			
AFS^a^		Significant reduction in the subscale school reluctance	*d* = −0.34	0.05
		No significant changes in the subscale test anxiety	*d* = −0.26	0.08
		No significant changes in the subscale general anxiety	*r* =.49	0.10
DISYPS^b^		Significant reduction in the subscale for social anxiety	*d* = −0.82	0.01
SUD^c^		Significant reduction in the subjective distress	*t* = 2.32	0.046
Biggs et al. [55]	45			
SUD^c^		Significant reduction in the scale for subjective distress in Session 1	$\:\beta\:$ = −0.40, *F*(1,449) = 140.9	< 0.001
		Significant reduction in the scale for subjective distress in Session 2	$\:\beta\:$ = −0.23, *F*(1,409) = 78.8	< 0.001
Kahlon et al. [59]	27			
PSAS^d^		Significant reduction in the scale for public speaking anxiety	*d* = 1.53	< 0.001
Kahlon et al. [56]	73			
PSAS^d^		Significant reduction in the scale for public speaking anxiety	*d* = 0.83	< 0.001
SPS-6^e^		Significant reduction in the scale for social phobia in the VR exposure groups; no sig. group differences	*d* = 0.22	0.007
SIAS-6^f^		No significant reduction in the scale for social interaction anxiety over time in the VRET groups, no significant group differences	$\:\beta\:$ = −1.53	0.20
Whiteside et al. [57]	20			
SUD^c^		Significant reduction in the scale for subjective distress	*F*(1,18) = 126.50, *d* = 2.08*	< 0.001

^a^ AFS: Angstfragebogen für Schüler (Questionnaire for Anxiety in Students) [60]; ^b^ DISYPS: Diagnostik-System für psychische Störungen nach ICD-10 und DSM-5 für Kinder und Jugendliche – III (Diagnostic System for Mental Disorders in Children and Adolescents – III) [61]; ^c^ SUD: subjective units of distress [65]; ^d^ PSAS: Public Speaking Anxiety Scale [64]; ^e^ SPS-6: Social Phobia Scale [66]; ^f^ SIAS-6: Social Interaction Anxiety Scale [66]. Statistical parameters: N = in analysis included sample size; *d* = Cohen’s *d*; *r* = correlation coefficient, $\:\beta\:$ = regression coefficient; *F* = *F*-statistic, *t* = *t*-statistic. * = result for both conditions (verbal and virtual exposure combined; no sig. difference between both conditions in subjective distress.)

### Meta-Analysis of subjective units of distress (SUDs): Pre-Post changes

To evaluate the effect of VR interventions on subjective distress, a random effects meta-analysis was conducted using the meta package [54], based on data from two studies [55, 57]. The meta-analysis focused exclusively on within group pre-post changes in subjective units of distress (SUDs). Between-group effects at post-test were not pooled due to differences in outcome measures and study designs.

A third study by Beele et al. [58] could not be included because means and standard deviations for individual timepoints were not reported; nevertheless, their findings are consistent with the results of the meta-analysis. Data from Biggs et al. [55] were split into two separate samples for analysis, reflecting data from two distinct VR exposure sessions, resulting in k = 3 studies in the meta-analysis. This decision was based on the within-subject crossover design of the study, where one half of the participants received the VR intervention in the first session and a verbal exposure in the second, while the other half received the conditions in reversed order.

Since lower scores on the SUD indicate improvement, effect sizes were inverted so that negative values reflect symptom reduction. Effect sizes were calculated using standardized mean differences (SMD; Hedges’ *g*), and variance estimates were derived based on within-group pre-post comparisons.

**Fig. 2:**
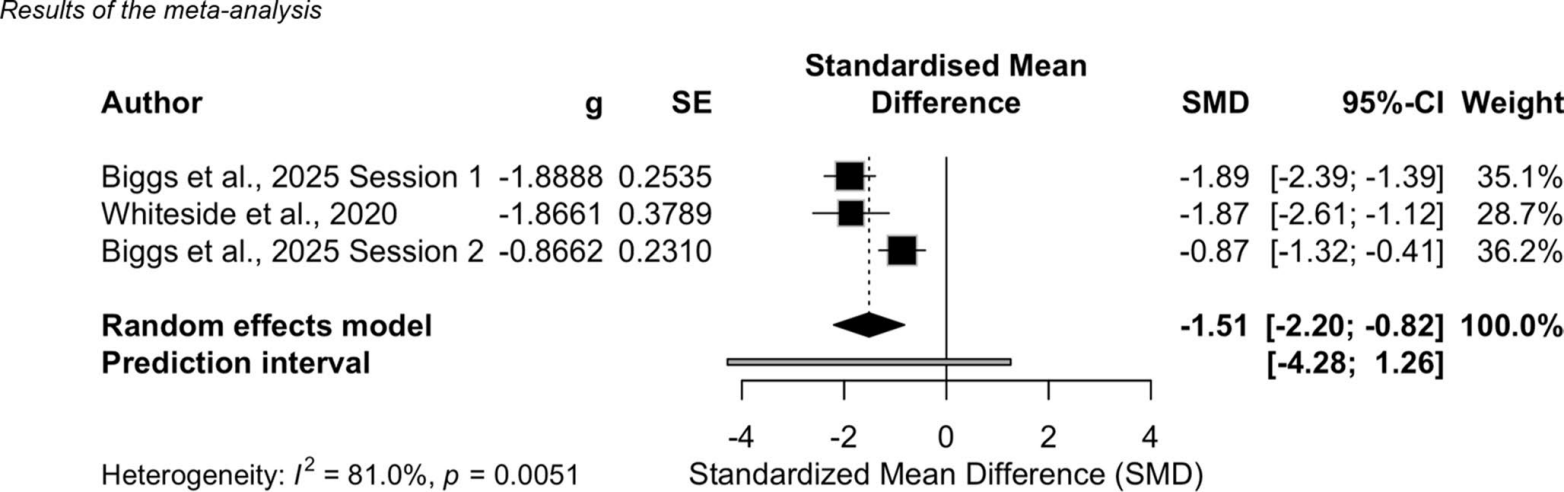
*Results of the meta-analysis. Note.* To ensure consistency in interpretation, negative effect sizes reflect reductions in anxiety or distress

The meta-analysis (see Fig. 2) revealed a statistically significant overall effect of VR exposure on subjective distress, with a large effect size of *g* = − 1.51 (95%CI [− 2.20, −0.82], *p* <.001), indicating a substantial reduction in distress. According to Cohen’s [67] benchmarks, this corresponds to a large effect. Heterogeneity across studies was assessed using Cochran’s Q test and quantified with *I*^2^ and $\:\tau\:$^2^. The test indicated significant heterogeneity (*Q*(2) = 10.55, *p* <.005), suggesting substantial variability in effect sizes across studies. These results indicate that study-specific factors, such as sample characteristics and VR intervention implementation, may have influenced effect sizes.

Despite the small numbers of studies samples (*k* = 3), the findings support the effectiveness of VR exposure in reducing subjective distress. Participants in Beele et al. [58] also reported significantly lower distress from the beginning to the end of VR sessions, with large within-session effects across all exposure situations, providing additional support for the benefits of VR-based interventions. However, the high heterogeneity warrants cautious interpretation.

### Effectiveness of VR exposure compared to other methods

Two of the included studies [55, 57] directly compared VR exposure to traditional in sensu exposure methods, which were either verbal or imaginal, using subjective units of distress (SUDs) as outcome measures in a within-study design. Both studies found no significant differences in reduction of subjective distress between VR and traditional exposure methods, indicating comparable effectiveness.

Similarly, Kahlon et al. [56] used the Public speaking Anxiety Scale (PSAS) as the primary outcome measure in a four-armed randomized controlled trial. Participants in the VR exposure conditions showed significantly greater reductions in PSAS scores compared to the waitlist control group. However, no significant differences were found between VR exposure and an online psychoeducation plus online exposure group.

Due to differences in outcome measures (SUD vs. PSAS), a quantitative synthesis across studies was not possible. Nevertheless, findings suggest that VR exposure is at least as effective as traditional exposure methods, supporting its potential as an alternative treatment option in children and adolescents.

#### Adherence

The average attrition rate (based on the five included studies), defined as the relative number of participants who started the intervention but did not complete all VR intervention sessions, was 13.3%, ranging from 0% to 27%, indicating that the interventions were generally well accepted and adhered to. 

#### User experience

User experiences were explored though non-standardized semi-structured interviews in the studies by Biggs et al. [55] and Whiteside et al. [57]. Participants described VR exposure as more realistic compared to traditional verbal or imaginal exposure but also reported that VR made them feel more anxious.

Higher levels of presence, the feeling of being there, were associated with increased state anxiety but did not significantly influence changes in test anxiety or general anxiety [see also Table 4;58].

Physical side effects of VR exposure were measured by Biggs et al. [55] and Whiteside et al. [57]. Biggs et al. [55] found higher levels of physical discomfort following VR exposure than after imaginal exposure. In contrast, Whiteside et al. [57] found no differences between virtual reality and verbal exposure. Across sessions, physical side effects decreased over time, suggesting habituation to the exposure environment independent of exposure type.

Regarding session length, VR exposure sessions were significantly longer than imaginal or verbal exposure sessions. In Biggs et al. [55], the mean duration of VR exposure in session one was 14.7 min (*SD* = 9.2) and of imaginal exposure 10.8 min.

(*SD* = 5.7), *p* =.007. In session two, the mean duration of VR exposure was 7.5 min (*SD* = 4.3) and of imaginal exposure 3.1 min (*SD* = 1.9), with *p* <.001. Whiteside et al. [57] observed similar findings, with VR exposure being significantly longer than verbal exposures (*F*(1,18) = 6.81, *p* =.018).

**Table 4:** Virtual reality (VR) interventions and user experience outcomes

Study and measures	VR user experience outcomes	Effect sizes/test statistics	*p*-values
Beele et al., [58]			
IPQ^a^	Higher levels of presence led to higher levels of state anxiety	*r* =.70	0.01
	No correlation between presence across sessions and post- and pre-differences in test anxiety	*r* = -.19	0.29
	No correlation between presence across sessions and post- and pre-differences in general anxiety	*ρ* = −0.29	0.21
Biggs et al., [55]			
SSQ-m^b^	Physical side effects were greater following VR exposure compared to verbal exposure (model-based effect)	*F*(1,43) = 9.8	0.005
	Decrease of physical side effects from first to last exposure session	*F*(3,114) = 8.0	< 0.001
User Experience Interview^c^	Participants rated VR exposure as more realistic compared to verbal exposure	*t*(40) = 5.56	< 0.001
	When participants described the VR exposure as unrealistic, then because the scenes felt inaccurate (scenes were different from reality) and because they were aware of the VR	–	–
Kahlon et al., [59]			
	–	–	–
Kahlon et al., [56]			
	–	–	–
Whiteside et al., [57]			
SSQ-m^b^	The physical side effects were lower at the end of the study (*M* = 1.24, *SD* = 0.30) than after VR exposure *(M* = 1.36, *SD* = 0.29) and after verbal exposure (*M* = 1.37, *SD* = 0.31)	–	all < 0.002
	The physical side effects did not significantly differ by exposure type	–	0.61
User Experience Interview^c^	VR made the participants more anxious (45%)	–	
	No statistical preference for one modality over the other	*t*(19) = 0.20	0.847
	Participants reported a preference for therapists who works with VR over verbal exposure	*t*(19) = 4.12	0.001

^a^ IPQ: Igroup presence questionnaire [68]; measures subjective sense of presence in VR.; ^b^ SSQ-m: Simulator Sickness Questionnaire [69]; assesses physical symptoms such as nausea, dizziness, eye strain; ^c^ User Experience Interview; semi-structured interview assessing realism, engagement, and subjective experience. Statistical parameters: r = Pearson correlation coefficient, *ρ* = Spearman correlation coefficient, *t* = *t*-test statistic, *F* = ANOVA *F*-statistic, *M* = mean, *SD* = standard deviation

## Discussion

### Principal findings

The findings of this review suggest that virtual reality (VR) exposure therapy is a promising and feasible intervention for reducing social and school-related anxiety symptoms in children and adolescents. Across the five included studies, reductions in subjective distress and anxiety symptoms were consistently reported with moderate to large effect sizes in public speaking anxiety and moderate improvements in school-related anxiety. Specifically, VR exposure therapy led to significant reductions in public speaking anxiety [56, 59], subjective distress [55, 57, 58], and social anxiety symptoms [58].

A meta-analysis of three effect sizes from two studies [55, 57] further supports these results. The analysis revealed a statistically significant large effect on VR exposure on subjective distress (*g* = − 1.51, 95%CI [− 2.20, − 0.82], *p* <.001). However, substantial heterogeneity was observed, indicating notable variability in effect sizes across study samples. The results underline the potential of VR-based interventions in reducing distress but also highlight the need for further research to explore factors contributing to differential effects across studies.

When directly compared to traditional exposure formats such as imaginal or verbal exposure, VR exposure showed comparable effectiveness. Both Biggs et al. [55] and Whiteside et al. [57] found no significant differences in subjective distress between VR and traditional exposure methods. Similarly, Kahlon et al. [56] showed that VR exposure led to greater symptom reduction than a waitlist control but found no significant differences when compared to online psychoeducation with online exposure. These findings suggest that VR exposure can be effective in reducing anxiety symptoms and may offer advantages such as immersive and controlled environments that enhance ecological validity and engagement. However, caution is warranted because comparison conditions varied across studies, and not all represent therapist-led, face-to-face exposure therapy.

Participants’ experiences with VR interventions were generally positive, with attrition rates being relatively low (average 13.3%). However, presence, the feeling of being there, was found to be associated with increased state anxiety during the VR experience [58], but did not influence long-term symptom reduction. User feedback, collected through semi-structured interviews, indicated that VR exposure was perceived as more realistic and anxiety-inducing than traditional methods [55, 57]. This heightened realism, although still only an approximation of real-life situations, could potentially enhance exposure efficacy but might also pose challenges for some users, particularly those with high anxiety.

Regarding physical side effects, results were mixed. Biggs et al. [55] reported greater physical side effects after VR exposure compared to imaginal exposure, while Whiteside et al. [57] found no such differences. Both studies observed a decrease in physical side effects over time, indicating that habituation may occur with more sessions. Additionally, the time spent in a VR exposure was significantly longer than that in a traditional exposure in both studies [55, 57]. This could partially explain the increased physiological responses in VR sessions that Beele et al. [55] found and also reflect the increased engagement with VR-based interventions.

Compared to adult populations, meta-analytic evidence similarly shows large effects of VR exposure versus waitlist controls [41, 43] and comparable efficacy to standard or imaginal exposure. These parallels suggest that VR-based interventions may be generalizable across age groups. However, heterogeneity in pediatric studies was higher, likely due to smaller sample sizes, variability in VR protocols, and developmental differences in engagement and anxiety responses. This underscores the importance of systematic evaluation in children and adolescents rather than assuming direct transferability from adult findings.

## Limitations, recommendations and future research

Overall, the reviewed studies support the feasibility and potential clinical utility of VR exposure therapy for children and adolescents with social related anxiety. However, several limitations restrict the generalizability and comparability of current findings. Two of the samples included in the meta-analysis were derived from the same study, and Kahlon et al., 2021 [56] was a pilot study for the subsequent main trial of Kahlon et al., 2022 [59], which should be considered when interpreting these findings. Further, heterogeneity in outcome measures, sample characteristics, and study designs across studies hinder the direct comparison and limit the possibility of conducting meta-analyses. To strengthen the evidence base, future studies should adopt standardized protocol, consistent anxiety measures, and comparable intervention designs. Additionally, the long-term effectiveness of VR exposure and the generalization of treatment effects beyond the VR setting remain largely unexplored. Most studies focus on immediate effects, leaving open questions of whether improvements can be maintained over time.

Evidence on potential side effects of VR interventions remains limited [46]. Few studies have systematically assessed physical symptoms such as nausea, dizziness, or eye strain, side effects that may particularly concern younger users. In a study of Tychsen and Foeller [70] with 50 children aged 4 to 10 years using a PlayStation VR headset for two 30-minute sessions, 94% completed both sessions without significant changes in visual or motor functions. Mild symptoms such as eye or head discomfort, fatigue, and motions sickness occurred in a few cases and only 6% discontinued early, suggesting that young children can generally tolerate immersive VR safely.

However, a challenge is, that most VR headsets are not designed to fit children’s smaller head and body sizes. As Whiteside et al. [57] noted, “the primary usability difficulty was the need to adjust the headset to fit each child”. The weight of popular headsets, around 515 g for the Meta Quest 3 [71] and 560 g for the PlayStation VR [72], must also be considered when planning session length, especially for smaller or younger children. Future research is urgently needed to assess potential side effects and establish best practices, as the “absence of evidence is not evidence of absence” [46].

Another open question concerns the optimal design of VR-based treatment in children and adolescents. While many VR interventions mirror traditional exposure therapy, the flexibility of VR offers new possibilities. For instance, integrating gamification elements could enhance motivation, reduce avoidance behavior, and improve adherence [73, 75]. This may be particularly beneficial for younger users who respond well to playful and interactive formats. Kahlon et al. [56] already incorporated various gamification elements into their VR exposure intervention, such as levels, star ratings, clear goals, feedback of the performance, varying difficulty levels, time constraints and offering choices for users to set their own task. Future research is needed to determine which gamification elements are most effective for children and adolescents and how many elements should be included to maintain motivation without compromising usability and therapeutic focus.

Looking ahead, the development of standalone VR exposure tools guided by virtual avatars could significantly enhance access to treatment. Such tools could support children while they are waiting for therapy, provide supplementary training between sessions, or serve as low-threshold preventive interventions for children with mild symptoms, potentially preventing symptom increase and chronification. Moreover, VR applications may empower therapists who are currently hesitant to conduct in-vivo exposure due to practical or emotional barriers [76], thereby broadening the implementation of exposure therapy in routine care.

Altogether, VR exposure therapy offers a promising and innovative addition to existing treatment options. When developed and implemented responsibly and based on empirical evidence, it has the potential to reduce the burden on mental health services and improve access to effective care, especially for children and adolescents who currently face long waiting times or limited treatment options.

## Conclusion

This systematic review highlights that virtual reality (VR) exposure is a promising approach for treating social-related anxiety in children and adolescents. Most studies found significant reductions in anxiety and distress following VR exposure, often with comparable effectiveness to traditional exposure methods.

Despite these encouraging results, the current evidence is still limited by methodological heterogeneity, a lack of long-term follow-up data, and unclear guidelines regarding safety, age-appropriateness, and optimal implementation. Future research should focus on standardizing intervention protocols, systematically investigating side effects, and further exploring the use of VR in younger populations.

Ultimately, VR exposure therapy holds considerable potential to complement traditional treatment approaches, expand access to care, and contribute to innovation in mental health services. With further research and technological advancements, VR could become a valuable and widely accepted tool in clinical practice.

## Data Availability

The data used for the meta-analysis are available from J. Baschab upon reasonable request.

## Appendix

**Table 5:** Quality assessment results of included studies using the mixed methods appraisal tool (MMAT) version 2018 [52]

Category of Study Designs	Methodological Quality Criteria	Beele et al., [58]	Biggs et al., [55]	Kahlon et al., [59]	Kahlon et al., [56]	Whiteside et al., [57]
Screening questions	S1. Are there clear research questions?	Yes	Yes	Yes	Yes	Yes
	S2. Do the collected data allow to address the research questions?	Yes	Yes	Yes	Yes	Yes
Quantitative randomized controlled trials	2.1. Is randomization appropriately performed?	-	Yes	-	Yes	Yes
	2.2. Are the groups comparable at baseline?	-	Yes	-	Yes	Yes
	2.3. Are there complete outcome data?	-	Yes	-	Yes	Yes
	2.4. Are outcome assessors blinded to the intervention provided?	-	Not reported	-	Not reported	Not reported
	2.5 Did the participants adhere to the assigned intervention?	-	Yes	-	Yes	Yes
Quantitative non-randomized controlled trials	3.1. Are the participants representative of the target population?	Yes	-	Yes	-	-
	3.2. Are measurements appropriate regarding both the outcome and intervention (or exposure)?	Yes	-	Yes	-	-
	3.3. Are there complete outcome data?	Yes	-	Yes	-	-
	3.4. Are the confounders accounted for in the design and analysis?	Partly fulfilled	-	No	-	-
	3.5. During the study period, is the intervention administered (or exposure occurred) as intended?	Yes	-	Yes	-	-
